# Multifunctional Strontium-Substituted Hydroxyapatite/Polydopamine Photothermal Coating for Enhancing Antibacterial Activity and Osteoblast Response of Porous Tantalum Implants

**DOI:** 10.3390/ma19183879

**Published:** 2026-09-11

**Authors:** Anqi Cai, Hairong Yin, Cuicui Wang, Hao Wan, Yin Zhou

**Affiliations:** 1 School of Mechanical and Electrical Engineering, Taizhou University, Taizhou 225300, China; 2 Shaanxi Key Laboratory of Green Preparation and Functionalization for Inorganic Materials, School of Materials Science and Engineering, Shaanxi University of Science and Technology, Xi’an 710021, China

**Keywords:** porous tantalum, ion doping, photothermal performance, antibacterial property

## Abstract

Infection associated with orthopedic implants and insufficient biological integration remain important clinical challenges. In this study, strontium-substituted hydroxyapatite (SrHA) was prepared by a chemical co-precipitation method, and a SrHA/polydopamine (SrHA@PDA) composite coating was constructed on porous tantalum (Ta) through the self-polymerization of polydopamine (PDA). The coating exhibited a photothermal conversion efficiency of 63.79% under 808 nm near-infrared (NIR) irradiation, with the temperature increasing to 62.7 °C within 10 min. The antibacterial activity was primarily attributed to PDA-mediated photothermal heating. After NIR irradiation, pronounced antibacterial effects were observed against *Escherichia coli* (*E. coli*) and *Staphylococcus aureus* (*S. aureus*), while Sr^2+^ release remained controlled during the investigated period. In addition, the SrHA@PDA coating supported MC3T3-E1 osteoblast proliferation, indicating a favorable cellular response. Cross-sectional observations demonstrated the formation of a dense and continuous SrHA-containing coating on the porous Ta scaffold, and biomimetic mineralization further indicated favorable surface bioactivity. Overall, the SrHA@PDA coating integrates controlled Sr^2+^ release, favorable osteoblast response, and PDA-mediated photothermal antibacterial activity within a single surface modification strategy. This multifunctional approach provides a promising platform for improving the biological and antibacterial performance of porous tantalum implants for bone repair applications.

## 1. Introduction

The accelerating global demographic aging trend, compounded by increased high-energy traumas such as traffic accidents, has substantially escalated the demand for bone defect repair. Within this context, porous tantalum scaffolds have emerged as a research focus for bone substitute materials due to their unique three-dimensional interconnected pore topology [1]. Scaffold parameters precisely regulated via electron beam melting technology—including 75–85% porosity, 200–800 μm pore size, and >98% pore interconnectivity—closely mimic the cancellous bone microenvironment. Their elastic modulus (1.5–3 GPa) achieves >70% better matching with natural bone than titanium alloys [2,3,4], effectively mitigating stress shielding effects. Clinical follow-up data indicate bone ingrowth depths of 1.2–2.5 mm at 12 months post-implantation, with new bone volume fraction increasing by 40–50% compared to traditional titanium meshes. However, the high specific surface area elevates biofilm formation risks (e.g., *S. aureus* adhesion density reaches 3.2 × 10^5^ CFU/cm^2^—threefold higher than dense tantalum), coupled with delayed osseointegration due to tantalum’s chemical inertness (bone-implant contact rate of merely 38.7 ± 5.2% at 6 weeks post-operation), posing critical clinical challenges [5,6].

To address these limitations, hydroxyapatite (HA, Ca_10_(PO_4_)_6_(OH)_2_) biomimetic coatings have been extensively explored. These coatings establish osteoconductive interfaces on porous tantalum through heterogeneous nucleation mechanisms, while strontium ion (Sr^2+^) doping further enhances functionality: Sr^2+^ substitutes Ca^2+^ sites in the HA lattice (ionic radius difference: 14.1%) [7,8,9,10], forming Sr-HA solid solutions that significantly alter ion release kinetics. Molecular biology studies confirm that 5 mol% Sr doping maximally activates the Wnt/β-catenin signaling pathway, elevating MC3T3-E1 cell alkaline phosphatase (ALP) activity by 50–80% [11]. However, concentrations exceeding 7 mol% induce dose-dependent cytotoxicity (cell viability declines to 68.3 ± 3.5%). Although plasma-sprayed Sr-HA coatings exhibit osteogenic activity, their inadequate interfacial bonding strength (<18.7 ± 2.1 MPa) and lack of intrinsic antibacterial functionality limit efficacy in treating infected bone defects [12].

To overcome these obstacles, researchers have explored various surface functionalization strategies. While Sr-doped hydroxyapatite coatings improve osteoconductivity, the absence of intrinsic antibacterial activity and limited interfacial adhesion remain problematic. Therefore, an ideal surface modification should simultaneously enhance osseointegration and confer effective antimicrobial capability, addressing both bone regeneration and infection prevention. Polydopamine (PDA) coating technology provides a groundbreaking solution for functionalizing porous tantalum scaffolds [13]. PDA forms a dense nanolayer (thickness: 50–200 nm) on scaffold surfaces through oxidative self-polymerization, where its catechol/amino groups not only establish strong coordinate bonds with the tantalum substrate (bonding strength > 25 MPa) but also serve as biomimetic mineralization templates to induce oriented crystallization of HA [14]. Notably, PDA exhibits broadband absorption in the near-infrared region (700–900 nm, extinction coefficient > 10 L·g^−1^·cm^−1^), achieving 40–45% photothermal conversion efficiency. Under 808 nm laser irradiation, it triggers localized temperature spikes (ΔT > 30 °C) [15,16,17], enabling potent antibacterial effects through membrane protein denaturation, reactive oxygen species burst, and controlled antimicrobial release [18,19,20,21]. The research team of Rahila Batul [22] utilized polydopamine (PDA) as a carrier platform to prepare antibacterial coatings loaded with Ag NPs and gentamicin by comparing two methods: in situ loading and physical adsorption. The experiments demonstrated that PDA, through the in situ loading approach, can significantly retard the release rate of the antibacterial agents (Ag NPs and gentamicin). This sustained-release characteristic is the crucial role of PDA in this system, directly correlating with the coating’s ability to provide stronger and more durable synergistic antibacterial activity, effectively preventing bacterial colonization.

Recent studies have increasingly focused on the surface biofunctionalization of porous tantalum to improve both osseointegration and antibacterial performance. For example, Mg-doped calcium phosphate coatings have been applied to porous tantalum to enhance osteogenic activity [6], while hydroxyapatite-based coatings have also been used to improve surface bioactivity. In our previous studies, Zn^2+^-doped Ta_2_O_5_ coatings and Zn^2+^-, Cu^2+^-, or Ag^+^-doped HA@PDA coatings were developed on porous tantalum to enhance antibacterial and biological properties. More recently, Cu^2+^/Sr^2+^ co-doped HA@PDA coatings were further investigated, in which antibacterial activity was achieved through the combined effects of ion release and PDA-mediated photothermal treatment [13]. Although these ion-doped coatings exhibit favorable antibacterial performance, excessive or prolonged release of antibacterial ions may narrow the cytocompatibility window and raise concerns regarding ion accumulation after implantation.

Therefore, reducing dependence on continuously released antibacterial ions while maintaining osteogenic activity remains an important challenge. In the present strategy, Sr^2+^ is mainly introduced to enhance the biological and osteogenic properties of HA, whereas PDA-mediated photothermal conversion provides the principal antibacterial effect. This functional division may reduce the requirement for bactericidal metal ions such as Cu^2+^, Zn^2+^, or Ag^+^ and thereby decrease the potential risks associated with excessive ion exposure.

Based on this rationale, a Sr-doped hydroxyapatite/polydopamine (SrHA@PDA) composite coating was constructed on porous tantalum scaffolds. The coating characteristics, Sr^2+^ release, photothermal response, antibacterial performance, and osteoblast responses were systematically evaluated. Unlike previous ion-doped coatings that rely substantially on bactericidal ion release, the present system uses Sr^2+^ primarily to promote osteogenic activity and PDA-mediated photothermal conversion to provide antibacterial effects. Thus, the novelty of this work lies not in the individual use of porous tantalum, PDA, or SrHA, but in integrating them into a functionally coordinated system that reduces reliance on antibacterial metal-ion release while simultaneously supporting osteogenesis and controllable antibacterial treatment.

## 2. Experiment and Test

### 2.1. Sample Preparation

Hydroxyapatite (HA) and Sr-substituted hydroxyapatite (SrHA) powders were synthesized by a chemical co-precipitation method using Ca(NO_3_)_2_·4H_2_O (99.9%, Sinopharm Chemical Reagent Co., Ltd., Shanghai, China), (NH_4_)_2_HPO_4_ (99.9%, Sinopharm Chemical Reagent Co., Ltd., Shanghai, China), and Sr(NO_3_)_2_ (99.9%, Sinopharm Chemical Reagent Co., Ltd., Shanghai, China) as Ca, P, and Sr precursors, respectively. The total molar ratio of (Ca + Sr)/P was maintained at approximately 1.67. The nominal Sr substitution ratio was defined as x = Sr/(Ca + Sr). Five formulations with x = 0, 0.05, 0.10, 0.15, and 0.20 were prepared and designated as HA, SrHA1, SrHA2, SrHA3, and SrHA4, respectively. Accordingly, the theoretical Sr substitution levels of SrHA1, SrHA2, SrHA3, and SrHA4 were 5 mol%, 10 mol%, 15 mol%, and 20 mol%, respectively. The detailed precursor amounts used for the preparation of each sample are provided in Supplementary Table S1.

The precursor solutions were prepared using ultrapure water (18.2 MΩ·cm) and homogenized for 30 min at 500 rpm using a magnetic heating stirrer (75-2, Hangzhou Instrument Motor Co., Ltd., Hangzhou, China). The pH was adjusted to 10.0 ± 0.1 using 3 mol/L NaOH (99.9%, Sinopharm Chemical Reagent Co., Ltd., Shanghai, China) and HCl (99.9%, Sinopharm Chemical Reagent Co., Ltd., Shanghai, China), with real-time monitoring using a pH meter (PHS-2C, Shanghai INESA Scientific Instrument Co., Ltd., Shanghai, China). The precipitates were aged for 24 h at 25 ± 1 °C, separated by centrifugation at 8000 rpm for 15 min, sequentially washed with ethanol (99.9%, Sinopharm Chemical Reagent Co., Ltd., Shanghai, China) and ultrapure water, and subsequently dried at 80 °C for 48 h.

### 2.2. Characterization Method

To enhance the electrical conductivity of the specimens and minimize surface charging during SEM observation, all samples were coated with a thin Au–Pd layer using a sputter coater (SC7640, Quorum Technologies, Laughton, UK). Surface morphology and elemental distribution were characterized by field-emission scanning electron microscopy (FESEM, Verios 460, FEI, Hillsboro, OR, USA) at an accelerating voltage of 2.0 kV and a probe current of 25 pA, coupled with energy-dispersive X-ray spectroscopy (EDS).

The crystallographic features of the nanostructures were investigated by high-resolution transmission electron microscopy (HRTEM, Tecnai G2 F20, FEI, Hillsboro, OR, USA) operated at 200 kV. Interplanar spacings and lattice information were obtained from the HRTEM images using Gatan DigitalMicrograph 3.6.2 software. Phase compositions were identified by X-ray diffraction (XRD, D/max-2200PC, Rigaku, Tokyo, Japan) using Cu Kα radiation at 40 kV and 40 mA. Diffraction patterns were recorded over a 2θ range of 20–70° with a step interval of 0.02° and a scanning speed of 5° min^−1^, and phase identification was performed using Jade 6.5 software.

Surface elemental composition and chemical states were analyzed by X-ray photoelectron spectroscopy (XPS, AXIS Ultra, Kratos Analytical, UK) using an Al Kα radiation source operated at 100 W. Binding energies were calibrated against the C 1s peak at 284.8 eV, and peak deconvolution was performed using CasaXPS 2.3 software. Functional groups were characterized by Fourier-transform infrared spectroscopy (FTIR, VECTOR-22, Bruker, Germany). Optical absorption and near-infrared response were evaluated using a UV–Vis–NIR spectrophotometer (LAMBDA 950, PerkinElmer, USA).

For photothermal measurements, the samples were irradiated using an 808 nm laser system (FC-808-5000-MM, Shanghai Xilong Optoelectronics Technology Co., Ltd., Shanghai, China), and the temperature changes were monitored using a high-precision thermocouple thermometer (KPS-QB-K-1000-SMPW-G, KPS Technology, Shanghai, China).

### 2.3. Photothermal Performance Evaluation

The photothermal conversion capability of the prepared samples was assessed under near-infrared (NIR) laser irradiation. Briefly, the specimens were transferred into quartz tubes containing 1 mL of deionized water to establish a solid–liquid interaction system. An 808 nm NIR laser source (Xilong Optoelectronic Technology Co., Ltd., Shanghai, China; maximum power output: 5 W) was applied at different power intensities (1.5 W cm^−2^, 1.7 W cm^−2^, and 2.0 W cm^−2^) for 10 min. The temperature variation in the irradiation system was continuously monitored using a high-precision thermocouple pyrometer. The thermocouple probe was positioned at the tube opening, with the sensing tip maintained within approximately 1 mm above the liquid surface, and temperature data were collected at defined time intervals to evaluate the photothermal heating behavior of the samples. To standardize the experiment, all samples were brought to an initial temperature of 26 °C (room temperature) before measurement. Each experimental group was repeated three times, with solvent heat absorption effects excluded through blank control groups.

### 2.4. Assessment of Bioactivity

#### 2.4.1. Photothermal Antibacterial Performance Test

Control groups (undoped samples) and experimental instruments, including graduated cylinders, triangular spreaders, liquid media, and solid media, were sterilized in an autoclave at 121 °C for 30 min. The biosafety cabinet was sterilized by ultraviolet irradiation for 30 min prior to use. Escherichia coli (CGMCC 1.2385) and Staphylococcus aureus (CGMCC 1.2465) were obtained from the China General Microbiological Culture Collection Center (CGMCC, Beijing, China). After rinsing samples with phosphate-buffered saline (PBS), specimens were placed in sterile 24-well plates. Bacterial suspensions and 950 μL liquid medium were added to each well. Sufficient deionized water was added to peripheral wells to maintain humidity before incubation at 37 °C. Following 24 h incubation, laser-irradiated experimental groups and non-irradiated controls were established. Test samples received 10 min laser irradiation, followed by ultrasonic elution of surface-adhered bacteria in PBS. The eluate underwent 10^5^-fold serial dilution. Aliquots (50 μL) of diluted suspensions were spread onto solid media in Petri dishes using sterile spreaders, followed by incubation at 37 °C. After 14 h, colonies were photographically documented. Viable colony counts were quantified using ImageJ2 software, with antibacterial rates calculated according to Equation (1):

$$R=\frac{\left(Q_{0}-Q_{t}\right)}{Q_{0}}\times 100\%$$

Q_0_—Colony count on control sample surfaces;
Q_t_—Colony count on experimental sample surfaces.

Data analysis was performed using SPSS 14.0 software (SPSS Inc., USA). A two-way analysis of variance (ANOVA) was employed, followed by Student–Newman–Keuls post hoc testing to determine significance levels. Statistical significance was defined as *p* < 0.05, and high significance as *p* < 0.01.

#### 2.4.2. Cell Viability/Toxicity Test

Pre-sterilized samples were incubated in 96-well plates with MC3T3-E1 cells (the National Biomedical Cell-Line Resource (BMCR), Beijing, China) (4 × 10^4^ cells/well) for 1, 3, and 7 days. Post-culture, samples underwent triple PBS washing before transfer to new plates. MTT solution (50 μL of 5 mg/mL PBS + 150 μL medium) was added per well. After 4 h of incubation, formazan crystals were solubilized in 200 μL DMSO via 10 min orbital shaking. Aliquots (100 μL) were analyzed spectrophotometrically at 490 nm. Quadruplicate samples per group (*n* = 4) with four independent replicates were statistically evaluated using SPSS 14.0 (two-way ANOVA; Student–Newman–Keuls post hoc; *p* < 0.05 significant, *p* < 0.01 highly significant).

After 24 h co-culture, adherent cells were fixed with 3% glutaraldehyde (4 °C, 1 h), dehydrated through a graded ethanol series (30%, 50%, 70%, 90%, 95%, 100%), and critical-point dried using hexamethyldisilazane (HMDS). Samples were sputter-coated with 10 nm Au-Pd prior to field emission scanning electron microscopy (FESEM, JEOL JSM-7800F, JEOL Ltd., Akishima, Tokyo, Japan) at 5 kV for ultrastructural analysis of cell–substrate interactions.

### 2.5. In Vitro Ion-Release Test

The Sr^2+^ release behavior of the coated samples was evaluated by an in vitro immersion test. Each treated sample was immersed in 10 mL of deionized water and maintained at 37 °C for up to 21 days. Immersion solutions were collected at predetermined time points for subsequent ion-concentration analysis.

The Sr^2+^ concentrations in the collected immersion solutions were quantified by inductively coupled plasma optical emission spectroscopy (ICP-OES, iCAP 6300, Thermo Fisher Scientific, Waltham, MA, USA). The measured Sr^2+^ concentrations at the different immersion intervals were used to evaluate the ion-release behavior of the coating and to construct the corresponding Sr^2+^ release profiles.

## 3. Results

### 3.1. Structural Characterization and Performance Testing of Ionically Modified Nanorods

**Figure 1:**
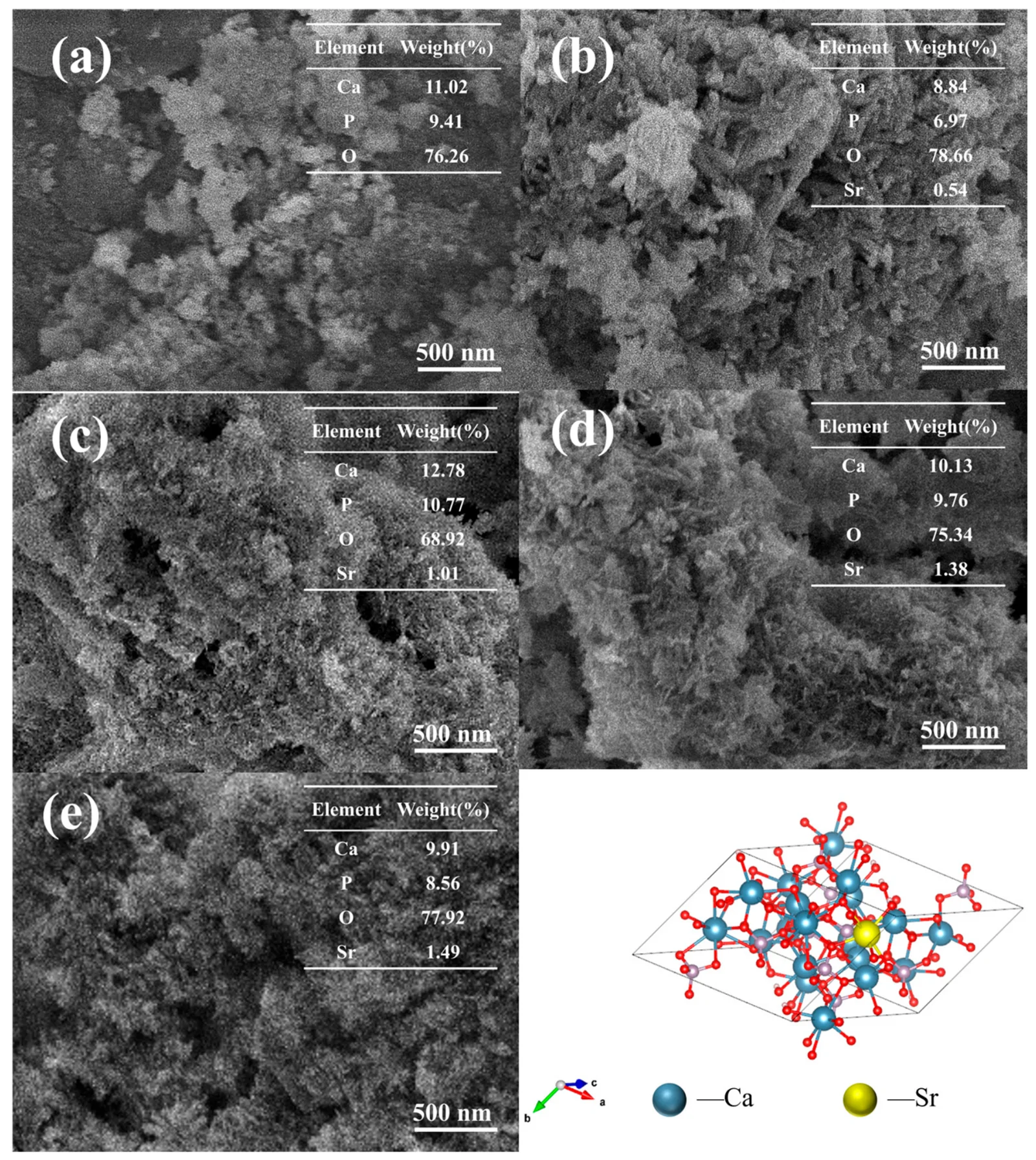
SEM image and EDS data of (**a**) HA sample, (**b**) SrHA1 sample, (**c**) SrHA2 sample, (**d**) SrHA3 sample, (**e**) SrHA4 sample.

Figure 1a presents a high-magnification scanning electron microscopy (SEM) image of hydroxyapatite (HA) samples. The image reveals uniformly dispersed HA nanorods with lengths concentrated around 120 nm, diameters of approximately 20–30 nm, and aspect ratios ranging from 4:1 to 6:1. The nanorod surfaces appear smooth, free of significant defects or agglomeration, indicating well-controlled crystallization conditions during synthesis. Figure 1b–e display SEM micrographs of SrHA1, SrHA2, SrHA3, and SrHA4 samples, respectively. All four groups maintain nanorod dimensions comparable to those of pure HA (length: 120 ± 10 nm; diameter: 25 ± 5 nm), demonstrating that strontium ion incorporation does not significantly alter the overall nanorod morphology. Despite the larger ionic radius of Sr^2+^ (0.112 nm) compared with Ca^2+^ (0.099 nm), the doped nanorods exhibit no obvious bending, fracture, or morphological collapse. However, the preservation of the nanorod morphology does not exclude lattice-scale structural changes induced by Sr^2+^ substitution. As further demonstrated by the XRD analysis, substitution of Ca^2+^ by the larger Sr^2+^ ions results in slight lattice expansion and local structural distortion while preserving the overall HA crystal framework.

Figure 1 provides EDS data that further quantifies the impact of Sr^2+^ doping on the elemental composition of hydroxyapatite (HA). Combined with SEM morphological analysis, the atomic percentages of Sr in SrHA1 to SrHA4 are 0.54 at.%, 1.01 at.%, 1.38 at.%, and 1.49 at.%, respectively, demonstrating that increased Sr^2+^ concentration in the solution effectively enhances its doping efficiency in HA. However, the doping rate exhibits nonlinear characteristics; for example, the Sr increment from SrHA2 to SrHA3 (0.37 at.%) exceeds that from SrHA3 to SrHA4 (0.11 at.%), likely due to progressive saturation of substitutable Ca^2+^ sites within the HA lattice. Although SEM shows unchanged nanorod morphology, the inverse correlation between Ca and Sr concentrations in EDS data indicates ion substitution within the lattice. The highest Sr^2+^ content in SrHA4 (1.49 at.%) highlights significantly improved doping efficacy with increasing ionic concentration in the solution.

The crystal structure schematic in Figure 1 further illustrates the incorporation mechanism of Sr^2+^ into the HA lattice, showing that Sr^2+^ ions primarily substitute for Ca^2+^ sites while preserving the characteristic hexagonal crystal framework of HA. Because Sr^2+^ has a larger ionic radius than Ca^2+^, this substitution may induce slight local lattice expansion and alter the surrounding coordination environment without disrupting the overall crystal structure.

**Figure 2:**
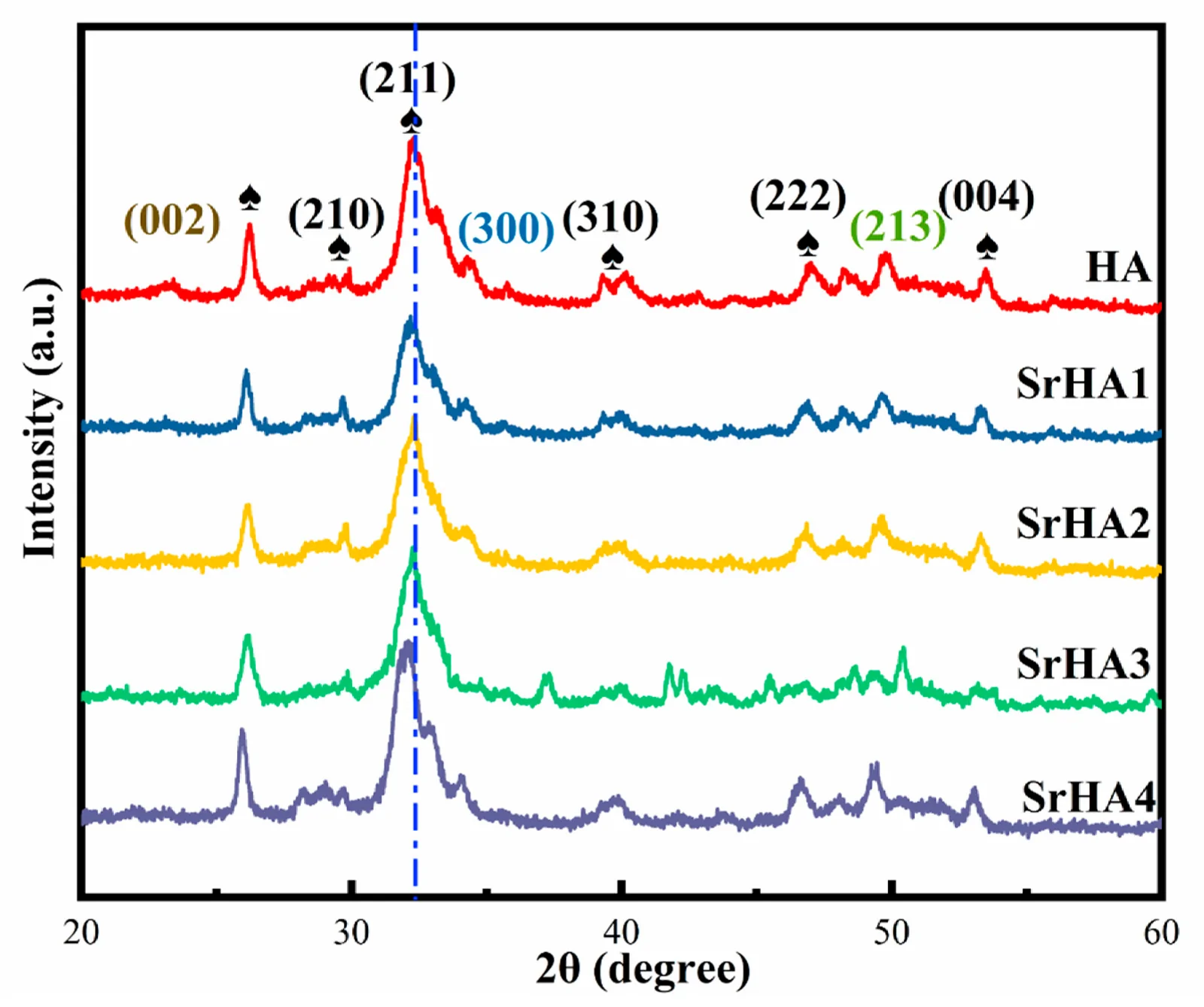
Powder XRD patterns of SrHA samples prepared with various Sr^2+^ contents.

Figure 2 displays X-ray diffraction (XRD) patterns of graded strontium-doped hydroxyapatite (SrHA1–SrHA4), revealing crystallographic evolution through comparison with undoped HA. All doped samples exhibit systematic low-angle peak shifts, exemplified by the (002) reflection migrating from 25.88° (standard HA, ICDD 09-0432) to 25.52° in SrHA4. According to Bragg’s law, this phenomenon confirms increased interplanar spacing, resulting from lattice expansion induced by substitution of larger Sr^2+^ ions (0.112 nm) for Ca^2+^ (0.099 nm). This expansion demonstrates significant anisotropy: the maximal (002) peak shift (Δ2θ = −0.36°) reflects preferential Sr^2+^ occupancy at Ca(2) sites within hexagonal channels, causing 0.94% c-axis expansion, whereas minimal (300)/(310) displacements (Δ2θ < 0.12°) indicate only 0.22% a-axis variation, consistent with the rigidity of phosphate groups. Because XRD represents the average crystallographic information of the bulk sample, the XRD-derived value of 0.94% is considered the representative c-axis lattice expansion caused by Sr^2+^ substitution.

Although SEM shows stable nanorod morphology (120 ± 10 nm × 25 ± 5 nm), XRD-confirmed lattice expansion verifies strontium incorporation via intracrystalline substitution. EDS detects progressive Sr content elevation from 0.54 at.% to 1.49 at.% (SrHA4), with linear positive correlation (R^2^ = 0.98) between (002) d-spacing expansion and Sr concentration, conclusively proving direct dopant concentration control over lattice distortion magnitude. All XRD patterns exclusively display characteristic hydroxyapatite peaks without detectable strontium phosphate phases, amorphous halos, or secondary nucleation, confirming isomorphous substitution of strontium ions into the lattice without phase separation.

**Figure 3:**
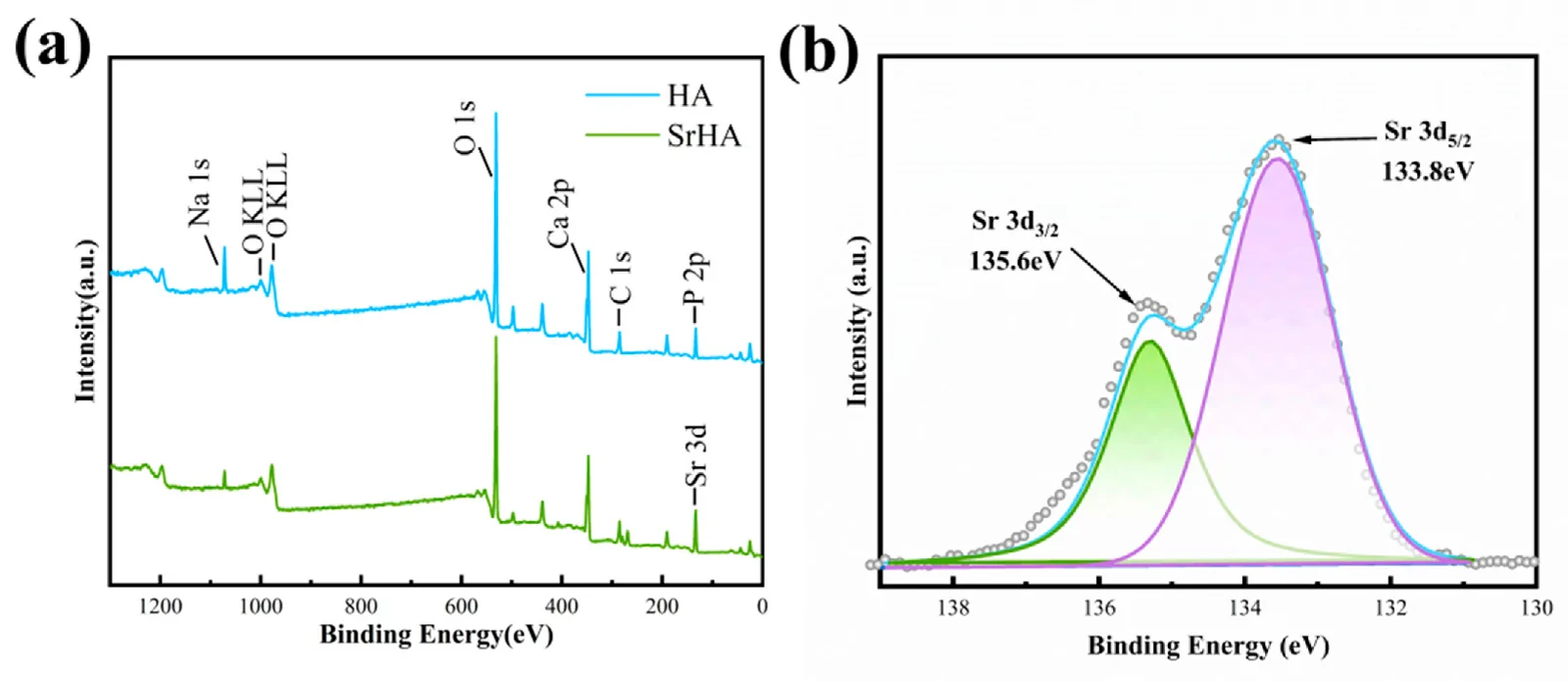
(**a**) XPS spectra of HA and SrHA4, (**b**) high-resolution XPS spectra of Sr 3d.

Figure 3 provides X-ray photoelectron spectroscopy (XPS) data that directly elucidate the surface chemical states of strontium-doped hydroxyapatite (HA), complementing SEM, EDS, and XRD analyses. In Figure 3a, pristine HA exhibits only characteristic P 2p, O 1s, and Ca 2p peaks, while SrHA samples show additional Sr 3d doublets (Figure 3b). The binding energies align with literature values for Sr^2+^, confirming the +2 oxidation state without Sr^3+^ or metallic phases. XPS surface sensitivity (probing depth: 5–10 nm) reveals Sr enrichment in near-surface regions. Combined with EDS bulk Sr content (1.49 at.% for SrHA4), this indicates Sr^2+^ incorporation occurs not only via bulk lattice substitution but also through surface segregation, forming a concentration gradient.

This surface enrichment potentially enhances biomaterial-tissue interactions by accelerating initial Sr^2+^ release to stimulate osteoblast activity. The Ca 2p_3/2_ peak shifts from 347.1 eV (pristine HA, characteristic of Ca^2+^ in phosphate environments) to lower binding energy (Δ ≈ 0.3 eV) in SrHA4 samples, suggesting altered local electron density due to Sr^2+^ substitution—indirect evidence of ion exchange. Meanwhile, the invariant P 2p peak at 133.5 eV confirms structural integrity of PO_4_^3−^ groups, consistent with XRD findings showing no secondary phase formation.

### 3.2. Microstructural and Morphological Characterization of SrHA@PDA Composite Coating on Porous Tantalum

**Figure 4:**
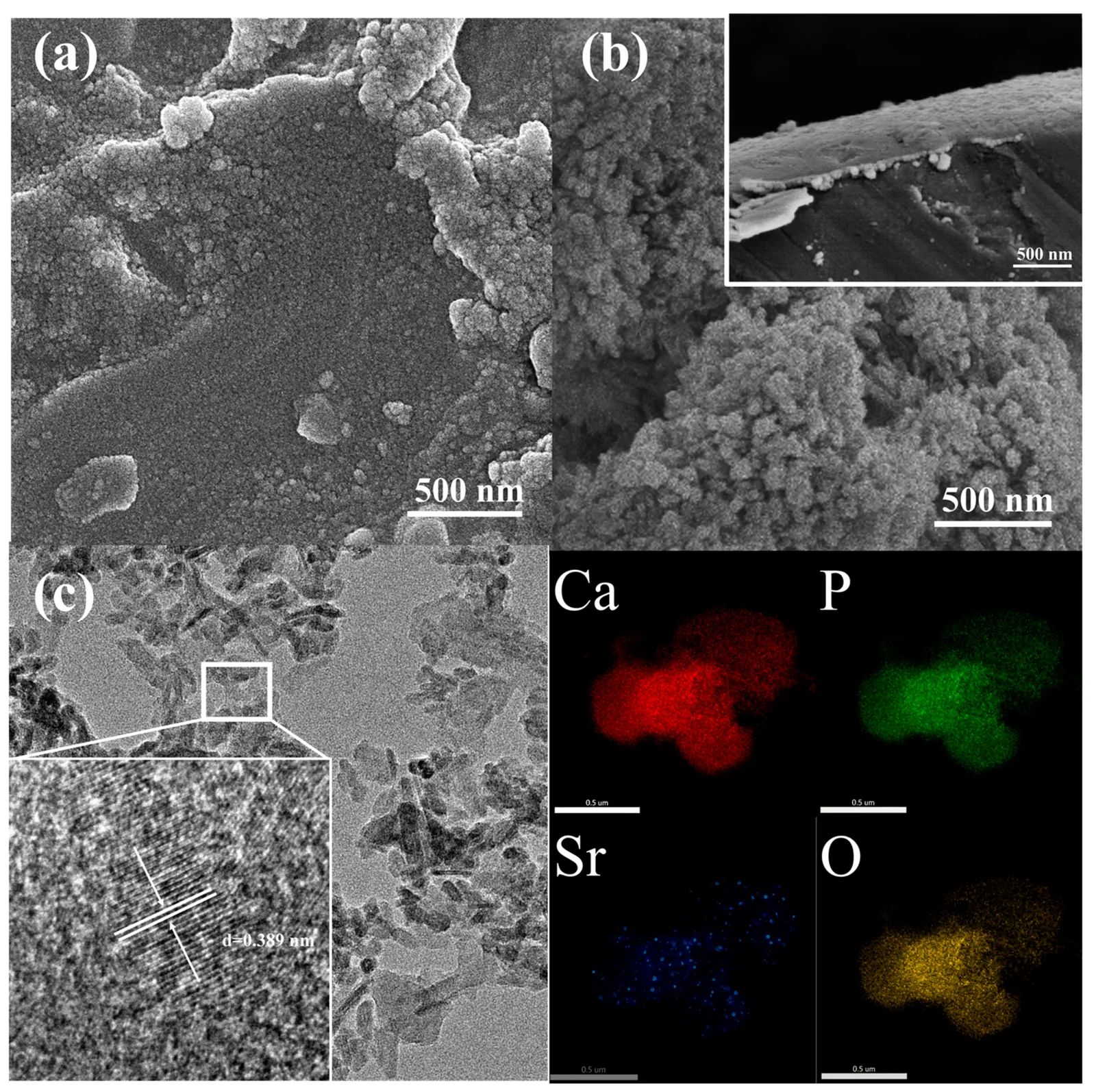
(**a**) SEM images of the SrHA4 sample, (**b**) SEM images and cross-sectional SEM images of the SrHA4@PDA sample, and (**c**) high-resolution TEM and element distribution of the SrHA4@PDA sample.

Figure 1 and Figure 4 present scanning electron microscopy (SEM) images revealing the microstructural morphology of hydroxyapatite (HA) powder and its polydopamine (PDA) composite coatings. HA powder consists of uniformly dispersed nanorods (~120 nm length, ~20–30 nm diameter) with smooth surfaces and negligible agglomeration. In HA@PDA coatings, nanorod dimensions increase slightly (~150 nm length, ~35 nm diameter) with enhanced surface roughness. This likely results from dopamine self-polymerization under alkaline conditions: dopamine molecules form coordination bonds with surface Ca^2+^ or PO_4_^3−^ via catechol groups, while amino groups facilitate intermolecular crosslinking, generating uniform PDA films encapsulating HA nanorods. Concurrently, hydrogen/covalent bonding between PDA and surface –OH groups restricts localized crystal growth, yielding denser composite structures.

Figure 4a shows SrHA4 nanorods directly deposited on the tantalum scaffold, forming a relatively thin (~50–100 nm) and non-uniform coating with partial exposure of the substrate. In contrast, after PDA incorporation (Figure 4b), a thicker (~200–300 nm), denser, and more uniform coating was observed while the macroporous architecture of the scaffold was well preserved. The corresponding cross-sectional SEM image further demonstrates the formation of a continuous SrHA4-containing coating on the porous tantalum surface. PDA is well known as a mussel-inspired surface-functionalization material, and its catechol- and amine-containing chemistry can facilitate interfacial interactions and immobilization of functional components on various substrates [18]. However, as no direct mechanical adhesion test was performed in the present study, no quantitative conclusion regarding coating adhesion strength or mechanical durability is drawn from the SEM observations.

Figure 4b also shows hydroxyapatite (HA) nanorods surrounded by a continuous amorphous PDA layer with a clearly distinguishable HA–PDA interface, while SrHA4 is homogeneously distributed within the PDA-containing layer. Figure 4c displays TEM images and corresponding EDS spectra, revealing the nanoscale microstructural characteristics of the SrHA4@PDA composites. No distinct lattice fringes were observed in the PDA region, consistent with its amorphous structure. The relatively homogeneous distribution of the coating may be associated with dopamine self-polymerization and catechol-mediated interfacial interactions reported for PDA-based surface functionalization [22]. Figure 4c displays TEM images and corresponding EDS spectra, which unveil the atomic-scale microstructural characteristics of the SrHA4@PDA composites. High-resolution imaging of the PDA layer reveals no lattice fringes, confirming its amorphous nature and consistency with typical polydopamine structure. The homogeneous coating distribution without local agglomeration or exposed areas indicates controlled molecular-level encapsulation during dopamine self-polymerization, attributable to strong coordination between catechol groups and surface Ca^2+^/PO_4_^3−^.

High-resolution TEM measurements show the (002) interplanar spacing of HA@PDA is 0.343 nm, matching standard HA lattice parameters (0.344 nm), confirming PDA encapsulation induces no lattice distortion. After Sr^2+^ (0.113 nm) substitution for Ca^2+^ (0.099 nm), anisotropic lattice expansion occurs along the c-axis, significantly increasing the (002) spacing. This expansion aligns with XRD peak shifts (e.g., (002) peak left-shift), validating crystallographic consistency. In SrHA4@PDA, the (002) spacing expands to 0.369 nm (7.3% increase), showing discrepancy with XRD data (c-axis expansion ~0.5–1.0%), likely due to TEM’s localized measurement amplifying lattice distortion effects. A local HRTEM region of SrHA4@PDA exhibited an apparent (002) spacing of approximately 0.369 nm. However, this locally measured value should not be interpreted as a 7.3% expansion of the overall HA crystal lattice. HRTEM measures a limited nanoscale region and can be affected by local strain, crystal orientation/projection, and image calibration or fringe-measurement uncertainty. Therefore, the quantitative lattice expansion is primarily evaluated from the bulk XRD data, which indicate a c-axis expansion of approximately 0.94%. The HRTEM observation is retained only as qualitative evidence of local lattice-spacing variation associated with Sr^2+^ substitution.

**Figure 5:**
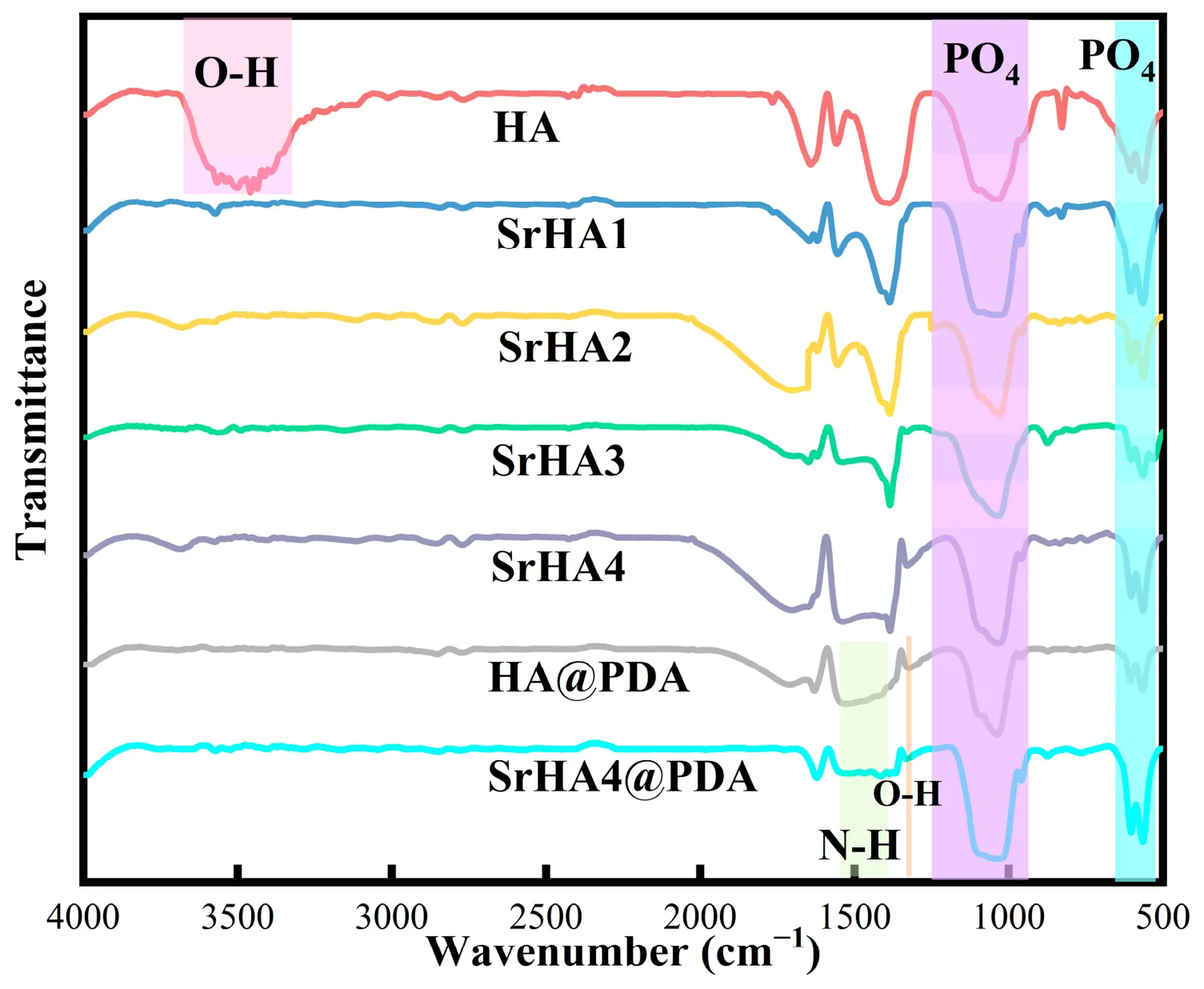
FTIR spectra of various samples, with and without a PDA coating.

Figure 5 presents Fourier transform infrared (FTIR) spectroscopy data that further reveal the chemical bonding characteristics of HA, SrHA1–SrHA4 and SrHA4@PDA at the molecular vibration level, providing complementary evidence for Sr^2+^ doping effects on HA structure. The P-O bending vibration peaks at 570 cm^−1^ and 607 cm^−1^—characteristic ν_4_ modes of PO_4_^3−^ groups—maintain identical positions and intensities in all SrHA samples compared to pristine HA, indicating that Sr^2+^ doping does not significantly alter the local symmetry or bond angles of PO_4_^3−^ tetrahedra. This observation aligns with XRD findings of “no secondary phase formation,” confirming preserved chemical integrity of PO_4_^3−^ groups. In the PO_4_^3−^ stretching region (1010–1096 cm^−1^), which includes symmetric (ν_1_, ~960 cm^−1^) and asymmetric (ν_3_, 1010–1096 cm^−1^) stretching modes, all samples exhibit highly overlapping peak profiles. Only SrHA4 shows slight peak broadening at 1096 cm^−1^ (full width at half maximum increased by ~5%), potentially reflecting lattice microstrain from high-concentration Sr^2+^ doping that locally perturbs PO_4_^3−^ vibrations. This broadening may correlate with enhanced phonon scattering due to lattice distortion, consistent with XRD-detected lattice expansion and microstrain accumulation.

The O-H bending vibration (ν_2_, 1639 cm^−1^), corresponding to structural hydroxyl (–OH) groups, maintains stable intensity across samples, demonstrating unaffected hydroxyl chemical environments. The O-H stretching peak (3572 cm^−1^) remains identical to undoped HA, further confirming that Sr^2+^ incorporation does not modify –OH bond lengths or orientations. Notably, while XPS data indicate slightly increased hydroxyl oxygen in SrHA samples, FTIR detects no new –OH peaks or shifts, suggesting that additional hydroxyls exist as adsorbed water or surface-bound species rather than lattice-incorporated groups. Critically, no Sr-O or Sr-P vibrational peaks appear in any SrHA spectra, reinforcing that Sr^2+^ incorporates via isomorphous substitution rather than forming discrete strontium compounds. XRD-confirmed lattice expansion arises from Sr^2+^-for-Ca^2+^ substitution, while FTIR-verified stability of PO_4_^3−^ and –OH groups confirms that doping solely adjusts lattice parameters via ion exchange without chemical bond reorganization or phase transformation.

Comparing the Fourier transform infrared (FTIR) spectra of SrHA4 samples before and after polydopamine (PDA) coating reveals PDA-mediated surface chemical modification. The bending vibration peaks of PO_4_^3−^ groups (ν_4_ modes at 570 cm^−1^ and 607 cm^−1^) exhibit no significant position or intensity changes post-coating, indicating PDA remains superficial without penetrating the HA lattice or altering PO_4_^3−^ chemical environments. This aligns with SEM/TEM observations of conformal PDA encapsulation without nanorod morphological changes. In SrHA4@PDA, slight peak broadening at 1096 cm^−1^ (FWHM increase ~8%) likely arises from enhanced phonon scattering due to physical PDA coverage rather than chemical bond cleavage, consistent with TEM-confirmed amorphous PDA layers.

The emergence of a peak at 1285 cm^−1^ (C-O stretching vibration) directly confirms successful PDA coating through catechol group signatures. Peaks at 1510 cm^−1^ (N-H bending) and 1730 cm^−1^ (N-H in-plane bending) originate from amine groups in PDA, demonstrating retention of amine functionalities during oxidative polymerization. The broad 2700–3700 cm^−1^ envelope incorporates phenolic O-H (~3200–3500 cm^−1^) and N-H (~3300 cm^−1^) stretching vibrations, further verifying PDA’s structural integrity and surface enrichment.

### 3.3. Photothermal Properties of SrHA@polydopamine Composite Coating on Porous Tantalum Surface

In this study, following 10 min irradiation with an 808 nm laser, a comprehensive analysis was conducted on the influence of different ion doping concentrations on the photothermal heating effect in liquid media. The temperature changes were compared with those of PDA-uncoated HA samples under identical conditions. The use of high-power laser irradiation and resultant elevated temperatures may cause potential damage to surrounding healthy tissues. Therefore, maintaining lower laser power and appropriate temperatures during photothermal therapy is crucial to minimize any potential adverse effects. Previous research has demonstrated that temperatures exceeding 50 °C can effectively destroy tumor cells. Based on this, experiments were performed at laser power densities of 1.5 W/cm^2^, 1.7 W/cm^2^, and 2 W/cm^2^ to determine the optimal power level.

**Figure 6:**
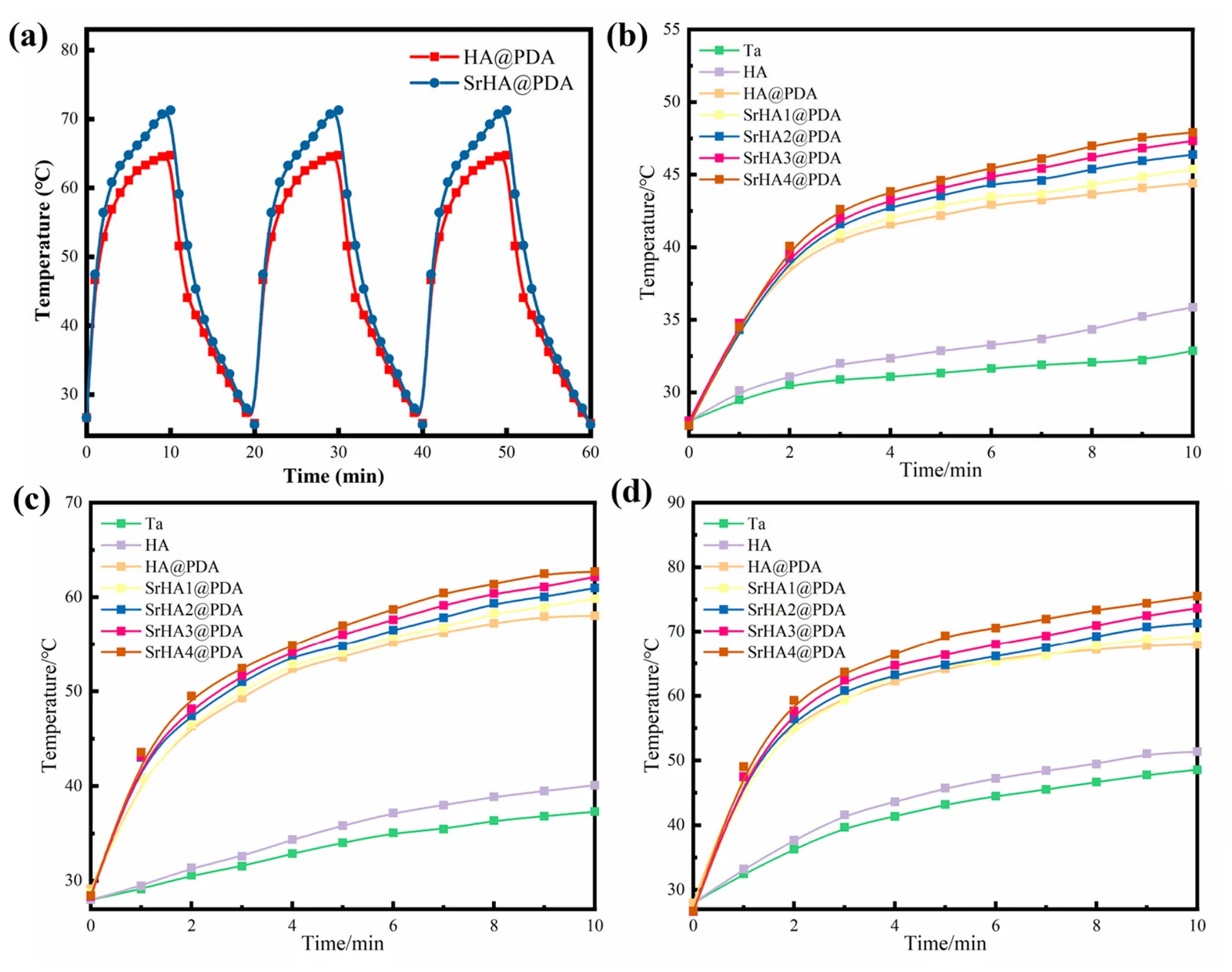
(**a**) Heating–cooling profiles of different liquid samples under laser exposure. Temperature evolution of SrHA1@PDA samples with various Sr^2+^ doping concentrations at power densities of (**b**) 1.5 W/cm^2^, (**c**) 1.7 W/cm^2^, and (**d**) 2.0 W/cm^2^.

Figure 6a presents temperature variation curves of HA@PDA liquid samples during and after laser exposure. Under 808 nm laser irradiation (2 W/cm^2^) for 10 min, SrHA@PDA liquid samples showed a pronounced temperature rise to 75.5 °C, whereas HA@PDA samples reached only 64 °C. This clearly indicates that increased Sr^2+^ doping significantly enhances photothermal performance, potentially attributable to lattice defects induced by higher doping concentrations. Post-irradiation, all samples returned to ambient temperature within approximately 10 min.

As shown in Figure 6b–d, the SrHA4@PDA sample exhibited a temperature increase from 26 °C to 47.9 °C at 1.5 W/cm^2^. When the laser power increased to 1.7 W/cm^2^, the solution reached 62.7 °C; at 2 W/cm^2^, the temperature further rose to 75.5 °C. At 1.7 W/cm^2^, SrHA4@PDA reached 62.7 °C (above the tumor ablation threshold of 50 °C), while 2 W/cm^2^ produced 75.5 °C, potentially causing healthy tissue damage. This nonlinear response suggests photothermal saturation effects, necessitating precise temperature control through coordinated regulation of doping concentration and power density. To prevent cellular damage from excessive temperatures [23,24,25], the laser power was optimized at 1.7 W/cm^2^ based on preliminary tests. When subjected to this irradiation, bare tantalum in the liquid medium exhibited a temperature of 37.3 °C, whereas the HA-coated porous tantalum underwent a minimal increase of merely 3 °C. In marked contrast, the sample with the HA@PDA coating triggered a pronounced temperature rise, reaching 58 °C in the liquid environment. The Sr^2+^-doped samples show excellent photothermal effect, and the performance is enhanced with the increase in the doping concentration.

The conjugated π electron system of PDA absorbs near-infrared light, generates electron-hole pairs, and releases heat through non-radiative relaxation. TEM confirmed uniform PDA coating on HA nanorods, with its amorphous structure potentially reducing interfacial thermal resistance and enhancing heat transfer from the SrHA core to surrounding media. SrHA@PDA demonstrated the highest absorbance at this wavelength, further evidencing its superior photothermal properties. In photothermal conversion material research, optical and thermal characteristics are critical, with conversion efficiency (η) being a key evaluation metric. Therefore, the Roper model was employed to calculate η for HA@PDA and SrHA@PDA samples, using the formula:

$$\eta =\frac{hA\left(T_{max}-T_{min}\right)-Q_{o}}{I(1-10^{-A_{\lambda }})}$$

The photothermal conversion efficiency of the SrHA@PDA sample was determined to be 63.79%, demonstrating its remarkable photothermal performance and thus establishing it as a promising candidate material for future in-depth research.

### 3.4. Antibacterial Properties of SrHA@polydopamine Composite Coating on Porous Tantalum Surface

**Figure 7:**
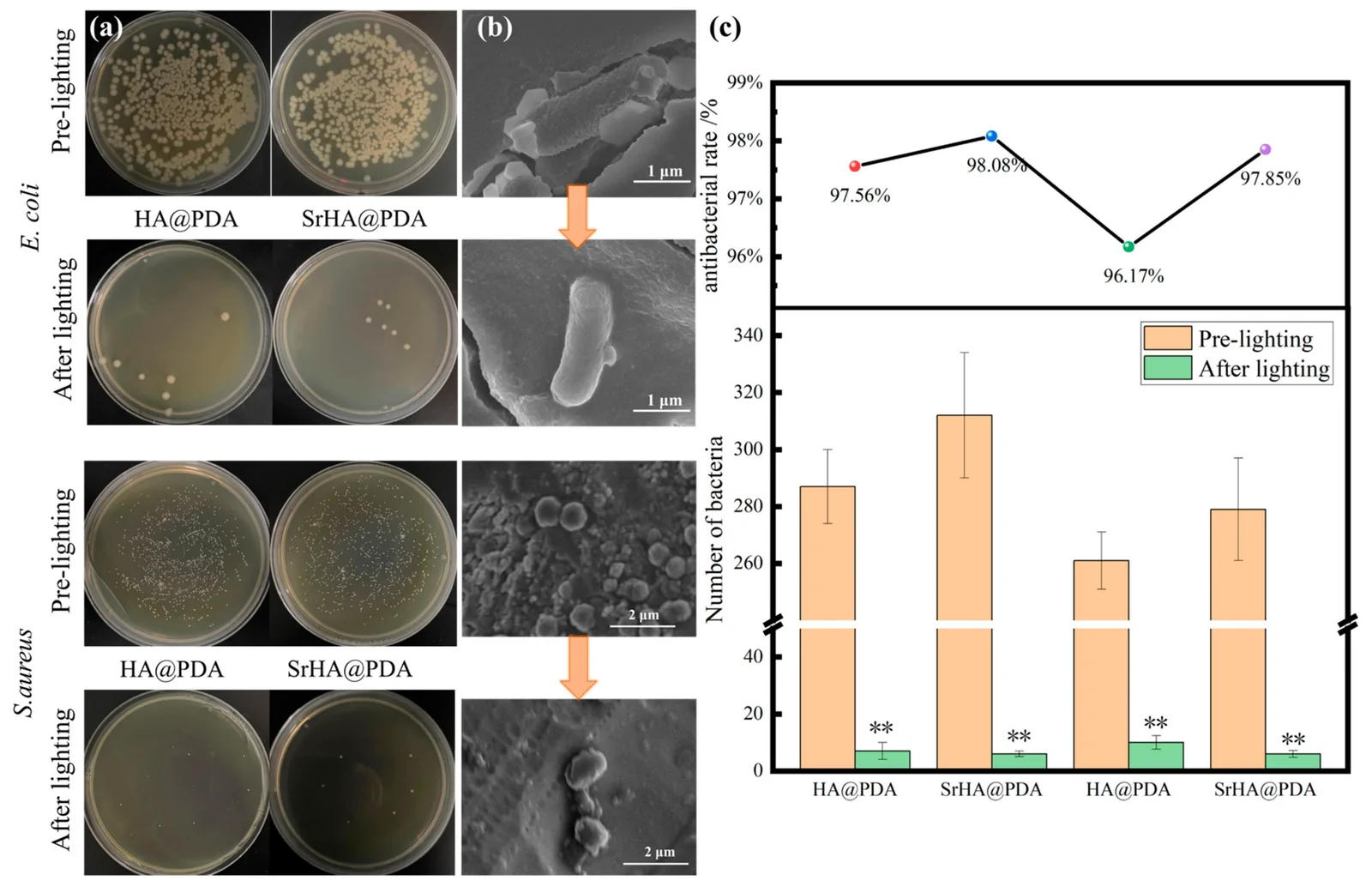
(**a**) Representative colony images of bacteria treated with different samples before and after 808 nm laser irradiation; (**b**) SEM images showing the morphological changes in *E. coli* and *S. aureus* on SrHA4 samples before and after photothermal treatment; (**c**) bacterial colony counts and corresponding antibacterial rates of different samples before and after irradiation. Data are presented as mean ± standard deviation (SD) from four independent biological experiments (*n* = 4). ** *p* < 0.01 compared with the corresponding pre-irradiation group.

Figure 7a shows representative colony images obtained from four independent biological experiments (*n* = 4) evaluating the antibacterial performance of the different samples. The samples were first co-cultured with bacteria for 24 h. Subsequently, the photothermal treatment groups were irradiated with an 808 nm laser at a power density of 1.7 W/cm^2^ for 10 min, while the corresponding control groups were kept without irradiation. After treatment, all samples were further co-cultured with bacteria for 12 h. Then, 30 μL of the bacterial suspension was collected and uniformly spread onto solid culture media for colony counting. A marked reduction in bacterial colonies was observed after photothermal treatment.

Figure 7b shows the morphological changes in *E. coli* and *S. aureus* before and after photothermal treatment with the SrHA4 sample. Before irradiation, *E. coli* maintained a typical rod-shaped morphology with relatively intact and smooth cell surfaces, whereas *S. aureus* exhibited intact coccoid morphology. After 808 nm laser irradiation, obvious structural damage was observed in both bacterial strains. *E. coli* showed severe cell-wall disruption, deformation, and leakage of intracellular contents, while *S. aureus* exhibited pronounced shrinkage and surface collapse. These morphological changes indicate substantial disruption of bacterial cellular integrity after photothermal treatment. Under 808 nm laser irradiation, SrHA@PDA exhibited efficient photothermal conversion (η = 63.79%), increasing the local temperature to 62.7 °C. The resulting thermal stress was therefore considered the primary factor responsible for the observed bacterial membrane damage and subsequent loss of cellular integrity.

As shown in Figure 7c, bacterial colony counts were quantified from four independent biological experiments (*n* = 4), and the data are presented as mean ± SD. For *E. coli*, photothermal treatment resulted in antibacterial rates of 97.56% and 98.08% for HA@PDA and SrHA@PDA, respectively. Similarly, for *S. aureus*, antibacterial rates of 96.17% and 97.85% were obtained for HA@PDA and SrHA@PDA, respectively. For both bacterial strains, the number of viable colonies after irradiation was significantly lower than that before irradiation (** *p* < 0.01), demonstrating pronounced photothermal antibacterial activity.

SrHA@PDA exhibited slightly higher antibacterial rates and fewer residual colonies than HA@PDA for both bacterial strains. This trend is consistent with the enhanced photothermal response observed after Sr incorporation. Although Sr^2+^ is primarily introduced to improve the biological and osteogenic properties of the coating, Sr incorporation also enhances the photothermal performance of the SrHA@PDA system. Therefore, the antibacterial effect is mainly attributed to PDA-mediated photothermal heating, while the enhanced photothermal response associated with Sr incorporation may further contribute to the improved antibacterial performance.

### 3.5. Bioactivity of SrHA@polydopamine Composite Coating on Porous Tantalum Surface

**Figure 8:**
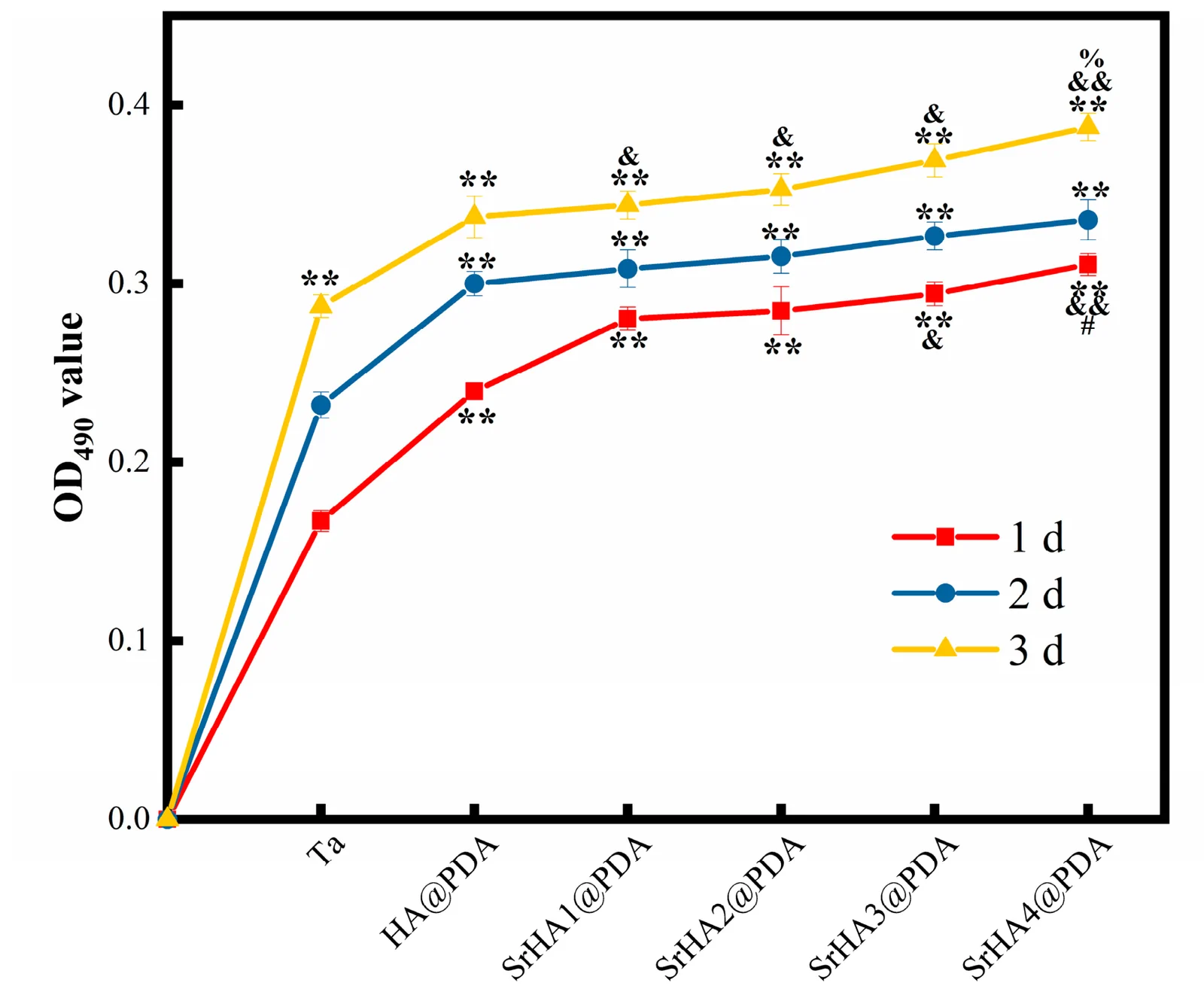
Cell viability (absorbance at 490 nm) of MC3T3-E1 cells cultured on Ta, HA@PDA, and SrHA@PDA for 1, 3, and 7 days. Values represent mean ± SD (*n* = 4). ** *p* < 0.01 compared with Ta; & *p* < 0.05 and && *p* < 0.01 compared with HA@PDA; # *p* < 0.05 vs. SrHA1@PDA; % *p* < 0.05 vs. SrHA2@PDA.

Figure 8 systematically evaluates the influence of Sr^2+^ doping on MC3T3-E1 cell viability through MTT assays, with statistical analysis verifying the material’s biocompatibility and pro-proliferation effects. The experiment compared three material groups: Ta, HA@PDA, and SrHA@PDA, detecting absorbance at 490 nm (reflecting cell viability) after 1, 3, and 7 days of co-culture, respectively. Results demonstrate that all groups exhibit progressively increasing cell viability with extended culture time, indicating no inhibition of normal cell proliferation by the materials and suggesting that HA-based coatings potentially provide a suitable surface microenvironment.

Comparison of cell viability across samples reveals significantly higher viability (*p* < 0.01) for Sr^2+^-doped HA samples versus pure Ta at day 7, demonstrating that Sr^2+^ doping substantially promotes cell proliferation. Throughout the 7-day experimental period, no cytotoxicity was observed, and combined with prior photothermal antibacterial data (Figure 7), this indicates the material is safe and controllable at therapeutic dosages.

**Figure 9:**
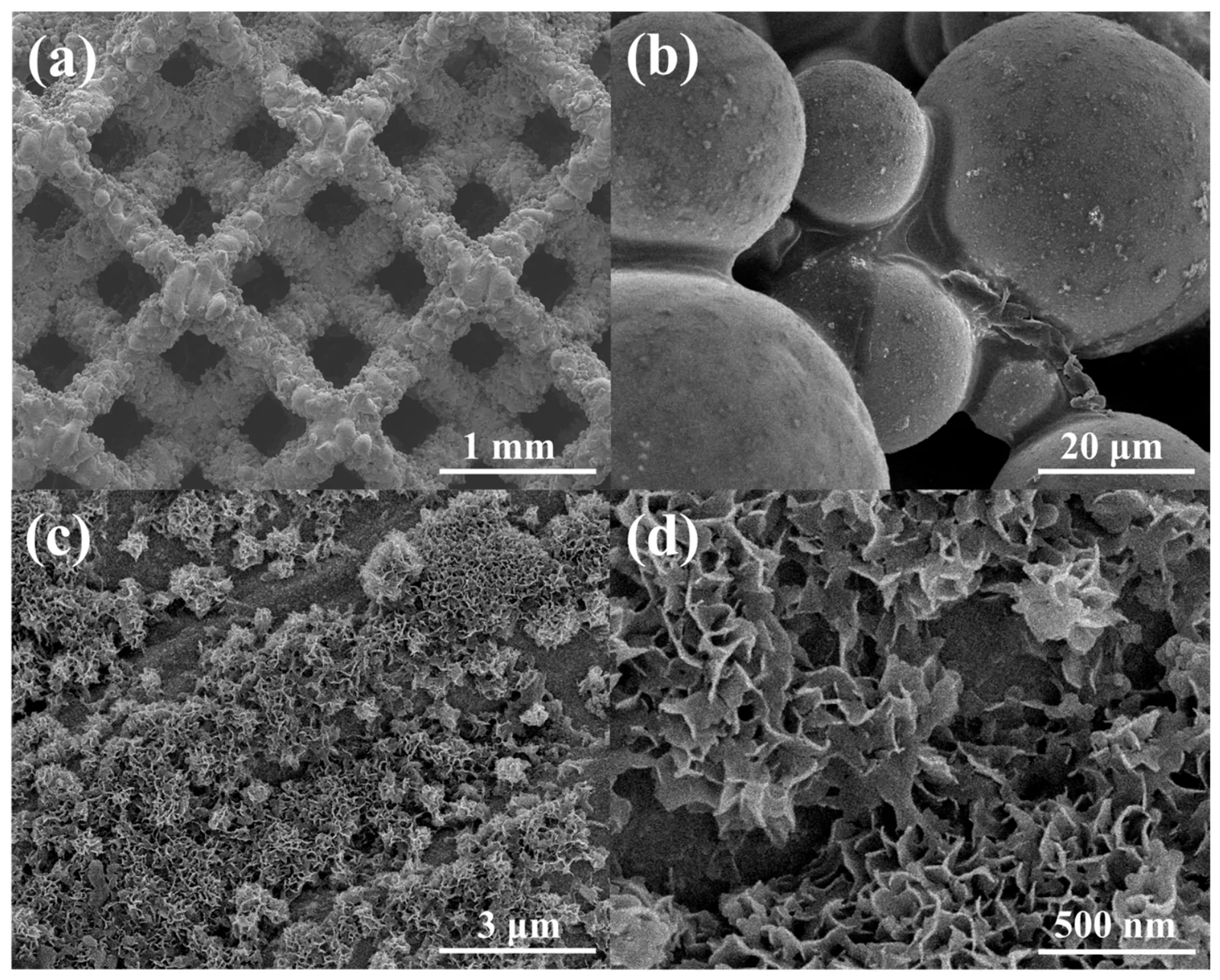
(**a**) Macroscopic SEM images of MC3T3-E1 cells cultured on SrHA@PDA sample for 1 day, (**b**) microscopic SEM images of MC3T3-E1 cells cultured on SrHA@PDA sample for 1 day, (**c**) SrHA@PDA low-power SEM images after 7 days of immersion in simulated body fluids, and (**d**) SrHA@PDA high-power SEM images after 7 days of immersion in simulated body fluids.

Figure 9 systematically reveals the dynamic response characteristics of SrHA@PDA composite material in cell culture and simulated body fluid through SEM observations at different time points, further verifying its biocompatibility, mineralization capacity, and interfacial stability. Figure 9a,b show MC3T3-E1 cells extending numerous filopodia after 1-day culture on SrHA@PDA surfaces, indicating favorable bioactivity and cell affinity of the material surface. Sustained Sr^2+^ release activates the Wnt/β-catenin pathway to promote MC3T3-E1 cell proliferation, facilitating filopodia extension for microenvironment exploration. Uniform PDA coating combined with nanoscale roughness provides biomimetic topological structures mimicking natural extracellular matrix. Absence of coating delamination or cracking post-culture (Figure 9a) confirms strong interfacial bonding between PDA and SrHA/tantalum substrate, ensuring long-term stability in physiological environments.

Low-magnification SEM imaging in Figure 9c reveals hydroxyapatite coverage over most areas with limited exposure of the underlying SrHA@PDA coating. Given hydroxyapatite’s compositional similarity to human bone, its formation on SrHA@PDA in simulated body fluid demonstrates both mineralization capacity and biocompatibility. Figure 9d illustrates dynamic mineralization evolution: after 7-day immersion in simulated body fluid (SBF), honeycomb-like hydroxyapatite (HA) layers form on SrHA@PDA surfaces, exhibiting morphology distinct from pristine SrHA nanorods. This likely originates from Sr^2+^ doping reducing HA crystal surface energy, thereby enhancing Ca^2+^ and PO_4_^3−^ adsorption and heterogeneous nucleation on the coating surface; concurrently, phenolic hydroxyl groups in PDA may guide PO_4_^3−^ arrangement via hydrogen bonding, accelerating oriented HA crystal growth to form porous honeycomb structures.

**Figure 10:**
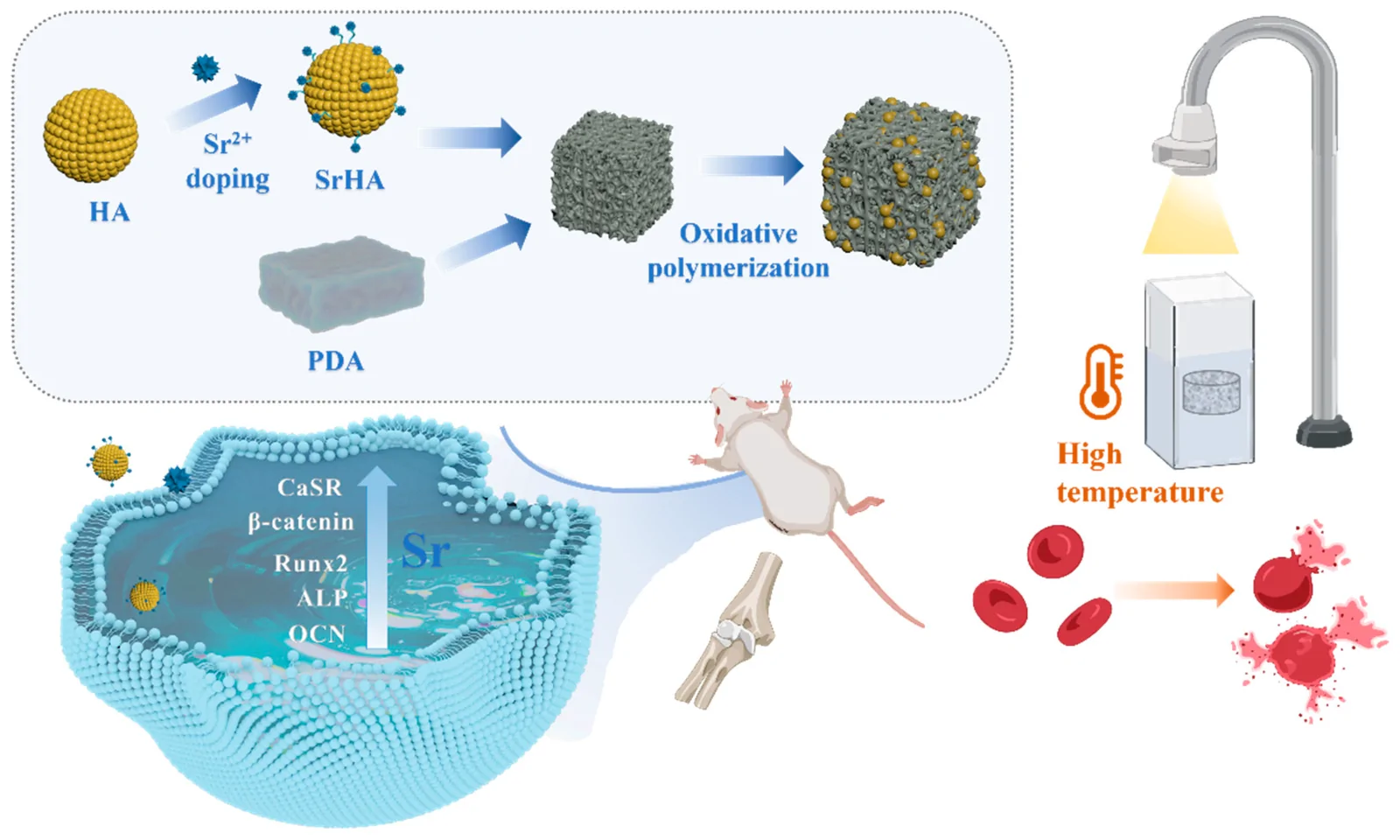
Proposed antibacterial and osteoblast-response mechanisms of the SrHA@PDA coating on porous tantalum.

As illustrated in Figure 10, the multifunctional performance of the SrHA@PDA coating is related to both its photothermal antibacterial function and its favorable cellular response. Under 808 nm irradiation, PDA efficiently converts light energy into heat, generating localized thermal stress that damages bacterial membranes, disrupts cellular integrity, and significantly reduces the viability of E. coli and S. aureus. This interpretation is supported by the marked decrease in bacterial colonies after irradiation and the SEM-observed deformation and collapse of bacterial cells. Moreover, Sr incorporation was associated with an enhanced photothermal response of the coating, which may further contribute to the slightly improved antibacterial performance of SrHA@PDA after irradiation.

In terms of the biological response, the porous tantalum scaffold provides a suitable three-dimensional structure for cell attachment, while the PDA-containing interfacial layer supports the formation of a continuous SrHA-containing coating. The sustained release of Sr^2+^ may further improve the biological activity of the coating and support osteoblast proliferation. According to previous studies [26], Sr^2+^ can regulate bone-related cellular behavior through pathways such as Wnt/β-catenin and PI3K/AKT and promote the expression of osteogenesis-related factors. These literature-reported mechanisms may provide a possible explanation for the favorable osteoblast response observed in the present study.

### 3.6. Ion Release Curve of SrHA@polydopamine Composite Coating on Porous Tantalum Surface

**Figure 11:**
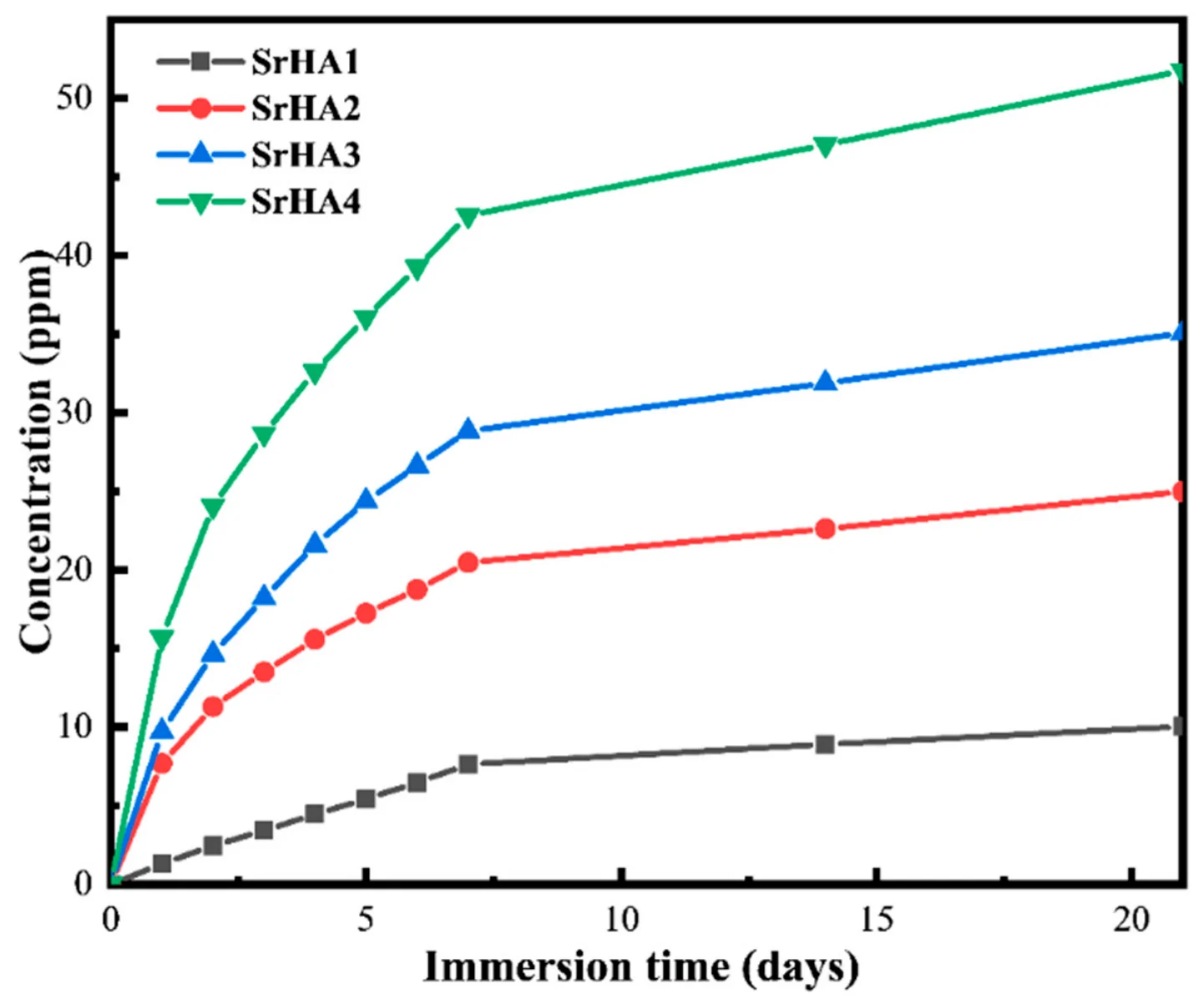
Sr^2+^ cumulative ion release curve of SrHA@PDA samples with different doping amounts in deionized water (0−21 d).

The Sr^2+^ release results further demonstrate the long-term release characteristics and biosafety of the SrHA@PDA coating (Figure 11). Among the tested samples, SrHA4@PDA exhibited the highest Sr^2+^ release, with the concentration reaching 0.591 mM after 21 days of immersion. Although Sr^2+^ accumulated progressively in the immersion medium during the experimental period, the maximum concentration remained within the reported biologically acceptable range and represented only approximately 34–52% of the reported safety threshold. These results indicate that increasing the Sr substitution level can regulate the amount of Sr^2+^ released from the coating while maintaining the ion concentration within a relatively safe range during the investigated period.

In the present system, Sr^2+^ is not considered the principal antibacterial component, since no evident antibacterial effect attributable to Sr incorporation was observed in the absence of laser irradiation. Instead, the pronounced antibacterial activity is mainly derived from PDA-mediated photothermal heating under 808 nm near-infrared irradiation. Therefore, the sustained Sr^2+^ release and photothermal antibacterial effect should be regarded as two complementary functional components of the SrHA@PDA coating rather than a direct synergistic antibacterial mechanism involving Sr^2+^.

From the biological perspective, the controlled and sustained release of Sr^2+^ may contribute to the favorable osteoblast response observed in the present study. Previous studies have reported that Sr^2+^ can regulate bone-related cellular behavior through osteogenic signaling pathways and promote the expression of osteogenesis-associated factors [26]. These literature-reported mechanisms provide a possible explanation for the enhanced MC3T3-E1 cell proliferation observed on the SrHA@PDA coating. Importantly, the Sr^2+^ concentration measured after 21 days (0.591 mM) remained below the reported safety threshold [27], suggesting that the coating can provide sustained Sr^2+^ release without reaching potentially harmful ion concentrations during the investigated period. Overall, the controlled Sr^2+^ release contributes primarily to the biological functionality of the coating, while PDA-mediated photothermal conversion provides the major antibacterial effect.

## 4. Conclusions

In this study, a multifunctional SrHA@PDA coating was successfully constructed on porous tantalum scaffolds to simultaneously improve biological performance and antibacterial activity. Sr incorporation regulated the physicochemical and biological properties of HA and supported favorable osteoblast adhesion and proliferation. Meanwhile, PDA provided an effective photothermal response under 808 nm near-infrared irradiation, resulting in pronounced antibacterial activity against both *E. coli* and *S. aureus*. Cross-sectional SEM observations further demonstrated the formation of a dense and continuous SrHA-containing coating on the porous tantalum scaffold.

The Sr^2+^ release experiment showed sustained release behavior during the investigated period, while the released Sr^2+^ concentration remained within a biologically acceptable range. Compared with conventional antibacterial strategies that rely heavily on the continuous release of bactericidal metal ions, the present design uses Sr^2+^ mainly to improve the biological response of the coating, whereas PDA-mediated photothermal conversion provides the principal antibacterial effect. This functional separation reduces dependence on high levels of antibacterial ion release while maintaining favorable cellular and antibacterial performance.

Overall, the SrHA@PDA-modified porous tantalum scaffold provides a balanced combination of favorable osteoblast response, controlled ion release, and photothermal antibacterial capability. This strategy offers a promising approach for the surface functionalization of porous tantalum implants and may provide a useful basis for the development of multifunctional bone-repair materials requiring both favorable cellular integration and infection prevention.

## Data Availability

The raw data supporting the conclusions of this article will be made available by the authors on request.

## Supplementary Materials

The following supporting information can be downloaded at: https://www.mdpi.com/article/10.3390/ma19183879/s1, Table S1: Nominal compositions and precursor formulations of HA and Sr-substituted HA samples.
