# Supplementary material for: Multifunctional Strontium-Substituted Hydroxyapatite/Polydopamine Photothermal Coating for Enhancing Antibacterial Activity and Osteoblast Response of Porous Tantalum Implants
Source: Materials (Basel). 2026 Sep 11;19(18):3879. doi: 10.3390/ma19183879 (PMC13608404; doi:10.3390/ma19183879)
Supplement: Supplementary file 1 [file materials-19-03879-s001.zip › materials-4528919-supplementary.pdf]

Supplementary materials

$$(10-X)Ca\left(NO_3\right)_2\cdot 4H_2O + 6\left(NH_4\right)_2 HPO4 + XSr\left(NO_3\right)_2 \rightarrow Ca_{(10-X)}Sr_X(PO_4)_6(OH)_2$$

Table S1. Nominal compositions and precursor formulations of HA and Sr-substituted HA samples.

Sample	$Ca(NO_3)_2{\cdot}4H_2O$ (g)	$(NH_4)_2HPO_4$ (g)	$Sr(NO_3)_2$ (g)	x = Sr/(Ca + Sr)	Nominal Sr substitution (mol%)
HA	5.91	1.99	0	0	0
SrHA1	5.60	1.99	0.27	0.05	5
SrHA2	5.32	1.99	0.53	0.10	10
SrHA3	5.02	1.99	0.80	0.15	15
SrHA4	4.72	1.99	1.06	0.20	20
